# A Proposed Hypothesis on Dementia: Inflammation, Small Vessel Disease, and Hypoperfusion Is the Sequence That Links All Harmful Lifestyles to Cognitive Impairment

**DOI:** 10.3389/fnagi.2021.679837

**Published:** 2021-04-29

**Authors:** Antoine M. Hakim

**Affiliations:** ^1^Brain and Mind Research Institute, University of Ottawa, Ottawa, ON, Canada; ^2^Division of Neurology, University of Ottawa, Ottawa, ON, Canada

**Keywords:** obesity, sedentary lifestyle, sleep insufficiency, inflammation, cerebral small vessel disease, cerebral blood flow, cognitive decline, a unifying hypothesis

## Abstract

There is growing consensus that certain lifestyles can contribute to cognitive impairment and dementia, but the physiological steps that link a harmful lifestyle to its negative impact are not always evident. It is also unclear whether all lifestyles that contribute to dementia do so through the same intermediary steps. This article will focus on three lifestyles known to be risk factors for dementia, namely obesity, sedentary behavior, and insufficient sleep, and offer a unifying hypothesis proposing that lifestyles that negatively impact cognition do so through the same sequence of events: inflammation, small vessel disease, decline in cerebral perfusion, and brain atrophy. The hypothesis will then be tested in a recently identified risk factor for dementia, namely hearing deficit. If further studies confirm this sequence of events leading to dementia, a significant change in our approach to this debilitating and costly condition may be necessary, possible, and beneficial.

## Introduction

Dementia is a growing problem. It impacts the individual, his or her family, and the society they live in. Certain lifestyles have been confirmed to contribute to dementia, but the steps leading from the harmful lifestyle to its cognitive impact have not always been clear. This review proposes a stepwise progression from three selected lifestyles to their cognitive impact. It also suggests that the same process links all harmful lifestyles to their negative impacts on memory function.

Dementia represents a significant personal, family, and social burden, and the number of people with dementia is rising in many parts of the world (Patterson, 2018). One major factor leading to this increase is the longer life expectancy seen in many regions (Beltrán-Sánchez et al., 2015). As a result, the number of people with dementia has doubled since 1990 (GBD 2016; Dementia Collaborators, 2019). In contrast, there is growing consensus in the literature that dementia is not an inevitable companion to old age (Qiu and Fratiglioni, 2018). Rather, cognitive decline appears more likely when certain lifestyles have been present (Laurin et al., 2001; Serrano-Pozo and Growdon, 2019; Smith, 2019). This realization has shifted the scientific and social discourse in dementia from the search for a therapy to promoting prevention of the condition. The 2020 report of the Lancet Commission identified twelve potentially modifiable risk factors for dementia including less education, hypertension, hearing impairment, smoking, obesity, depression, physical inactivity, diabetes, and low social contact (Livingston et al., 2020), and suggested that 40% of worldwide dementias may be due to these factors.

Research has also provided two additional important observations relevant to the etiology of dementia. The first was that drugs that successfully eliminated cerebral accumulations of beta amyloid have so far shown only modest impact on cognitive deficits (Oxford et al., 2020), although trials are still ongoing. Ever since the original description that these proteins were present in the brains of individuals dying with dementia, they were considered to be etiologically significant in inducing dementia, and the modest impact they have had to date has forced a reappraisal of our approach to dementia.

The second landmark observation was that a decline in cerebral blood flow (CBF) was an early cerebral event that heralded the decline in cognitive function and may precede the appearance of the clinical syndrome by many years (Iturria-Medina et al., 2016). This finding confirmed that vascular insufficiency is a major etiologic factor that anticipates the onset of cognitive deficits, and that the protein deposits found in the brain of demented individuals were more likely a consequence of the disease rather than its cause. While this was a major step forward in our understanding of the etiology of dementia, it left open the question: do all harmful lifestyles lead to cerebral hypoperfusion? If so, what are the physiological mechanisms that lead to the decline in CBF when a harmful lifestyle has persisted?

**FIGURE 1:**
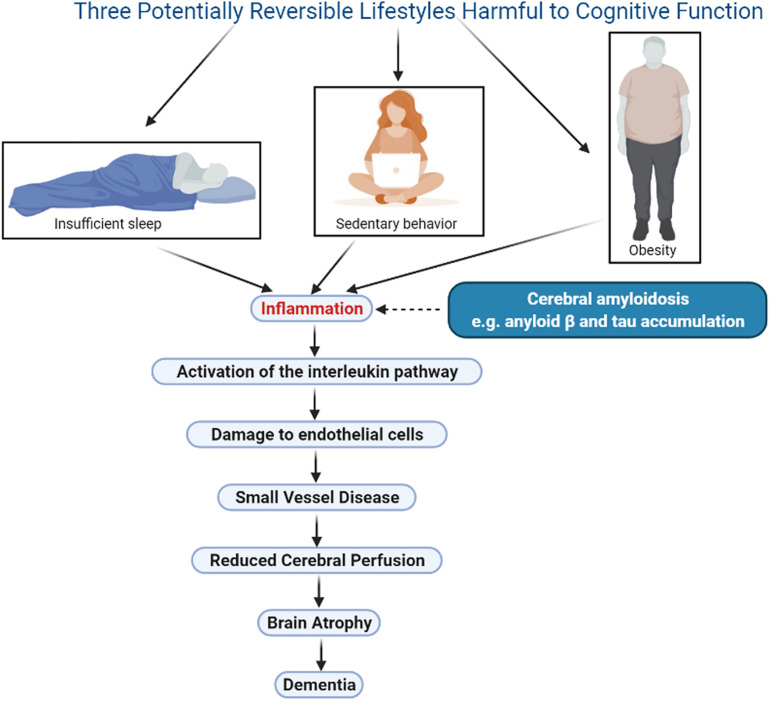
Illustration of a unifying hypothesis that proposes lifestyles that negatively impact cognition do so through the same sequence of events: inflammation, activation of the interleukin pathway, small vessel disease, damage to endothelial cells, decline in cerebral perfusion, and brain atrophy. Three harmful lifestyles are shown, obesity, sedentary behavior and insufficient sleep, all of which are potentially reversible therefore offering an opportunity for meaningful therapeutic interventions. Cerebral amyloidosis may play a synergistic role in this sequence however this phenomenon is currently not reversible. Figure was made in BioRender.com.

Several publications have proposed that inflammation may be the link between lifestyle, genetics, and Alzheimer’s disease (Uzoni et al., 2015; Newcombe et al., 2018), but the mechanisms that link inflammation to this outcome are not clear. This review will focus on 3 lifestyle factors that negatively impact cognition, namely obesity, sedentary behavior, and insufficient sleep. In each case, a summary of the research associating the lifestyle to subsequent cognitive decline will be presented, and the impact of the lifestyle on cerebral vascular perfusion will be explored. The potential mechanisms linking the lifestyle to its eventual impact on perfusion will then be reviewed. A unifying hypothesis will be proposed, namely, that all lifestyles that negatively impact cognition do so through the activation of inflammatory factors, which then lead to small vessel disease, resulting in a reduction in cerebral perfusion and causing atrophy of structures essential for normal cognition (Figure 1). The evidence supporting this hypothesis for the three chosen lifestyles will be presented, and its potential application in the setting of hearing deficit, a newly identified risk factor for dementia, will then be explored. As well, the implications of this renewed understanding of dementia for the individual and for society will be presented.

## Obesity

After some debate on the matter, it is now well accepted that obesity is associated with cognitive decline (Whitmer et al., 2008). The debate was triggered by a report that obesity may in fact reduce the risk for dementia (Qizilbash et al., 2015), but a number of methodological oversights were subsequently identified in this publication, pointing especially to the fact that a reduction in weight may occur in those affected by dementia (Singh-Manoux et al., 2018), implying that lower weight in later life may be due to the illness and consequently cannot be used to describe the association of obesity with dementia.

Studies recognizing that obesity in early and middle years negatively impact later cognitive abilities leave no doubt as to the long-term impact of obesity on memory functions, reestablishing obesity as a major risk factor for dementia (Xu et al., 2011; Kerti et al., 2013; Schwartz et al., 2013; Martin and Davidson, 2014).

The importance of this association is partly due to the growing problem of obesity in many jurisdictions. In the United States, in 2010, 35.5% of men and 35.8% of women satisfied the criteria for obesity (body mass index (BMI) of 30 or more) (Flegal et al., 2012), with a clear trend for increasing prevalence of obesity between 1999 and 2012 in both men and women (Ford et al., 2014).

The presence of inflammatory mediators in obesity is so well established that adipose tissue is now considered to be an immune organ (Makki et al., 2013). In their extensive review on the topic of inflammation in obesity, Chait and Hartigh (2020) point out that inflammation associated with adipose tissue is chronic, sterile, low grade and impacts function of liver, muscle, and pancreas. Visceral adipose tissue is associated with secretion of inflammatory cytokines, and the elevated levels of C-reactive protein (CRP) and serum amyloid A are likely in response to the interleukin-6 (IL-6) secretion from the adipose tissue (Gu et al., 2018b). As well, tumor necrosis factor alpha (TNFα) levels positively correlate with adiposity, BMI, insulin levels, and insulin resistance (Zahorska-Markiewicz et al., 2000; Kern et al., 2001).

A positive feedback loop may link inflammation and obesity. Genetic polymorphisms near a central regulator of inflammatory cell function that coordinates inflammation is associated with obesity in humans, and silencing it in the laboratory setting reduces obesity (Karunakaran et al., 2020). This points to the complex relationship between inflammation and obesity.

Recent literature emphasizes the fact that small vessel disease is a consequence of systemic inflammation (Gu et al., 2018a). Small vessel disease (SVD) refers to any pathologic process that damages small end arteries, arterioles, venules, and capillaries (Poggesi et al., 2012). Rouhl et al. (2012) confirmed the activation of an inflammatory process in 163 patients suffering their first-ever lacunar stroke, and this was followed a few short years later by a study in patients who suffered traumatic brain injury which confirmed that acute cerebral blood flow was a biomarker of underlying neuroinflammatory pathology (Sankar et al., 2019). Thus, inflammatory mediators are mechanistically associated with both SVD and reduced CBF.

Vermeer et al. (2007) included obesity in the list of lifestyles that are associated with SVD in the brain, evidenced on MRI by white matter hyperintensities. This association has been confirmed by other investigators (Yamashiro et al., 2014). Elevated BMI is also known to be associated with decreased blood flow in the prefrontal cortex of otherwise healthy adults (Willeumier et al., 2011), and a more recent evaluation using single photon emission computed tomography (SPECT) showed that higher BMI correlated with decreased perfusion in virtually all brain regions (Amen et al., 2020). Perhaps not surprisingly, given these facts, obese individuals had lower total cerebral brain volume compared to metabolically healthy non-obese individuals (Angoff et al., 2019), and Hamer and Batty (2019) reported that higher levels of all obesity measures were related to lower gray matter volume in many brain regions, an observation confirmed by Dekkers et al. (2019). Stanek et al. (2011) reported that a “(BMI X age”) interaction was noted in the corpus callosum, implying that damage to certain brain structures increases with both the degree of obesity and its duration.

In conclusion, obesity as a risk factor for cognitive decline would seem to fit well into the working hypothesis proposed here, namely that a specific physiological sequence, triggered by inflammation and resulting ultimately in brain atrophy, underlies the progression from the risk factor to the eventual cognitive decline.

## Sedentary Lifestyle

Sedentary behavior refers to activities that do not substantially increase energy expenditure above the resting level (Pate et al., 2008). In 2011, Barnes and Yaffe (2011) projected the impact of risk factor reduction on the prevalence of dementia and proposed that in North America, 21% of dementias could be attributed to physical inactivity. More recently, a study in Swedish women showed that 32% of those with low peak fitness, 25% with medium and 5% with high fitness subsequently developed dementia (Horder et al., 2018). In a systematic review and meta-analysis, Sanders et al. (2019) suggested a dose-response relationship between exercise and cognitive function, confirming the conclusion from a 10-year follow-up study by Hamer et al. (2018) which showed that memory and executive function were preserved by physical activity.

There is abundant literature confirming that sedentary behavior is associated with an inflammatory environment, and that exercise has anti-inflammatory benefits. After adjusting for all potential extraneous factors, sedentary behavior was associated with higher levels of TNFα and leptin (Allison et al., 2012). Henson et al. (2013) showed that sedentary time was detrimentally associated with levels of CRP, IL-6, and leptin. This was followed by a study in which reallocating 30 min of sedentary time to moderate and vigorous physical activity was associated with a more favorable inflammatory profile, characterized by higher adiponectin and lower complement component C3, leptin, and IL-6 (Phillips et al., 2017). Parsons et al. (2017) confirmed in the same year that sedentary activity was associated with higher levels of IL-6, CRP, and tissue plasminogen activator (tPA), while higher total physical activity levels were associated with lower levels of these inflammatory mediators. In 2018, Tsai et al. (2017) reported that an acute bout of aerobic exercise significantly increased serum levels of brain derived neurotrophic factor (BDNF) and insulin-like growth factor 1 (IGF-1), which may counter the inflammatory environment.

The literature linking prolonged sitting with a decline in cerebral perfusion is extensive. Carter et al. (2018) reported a significant decline in blood flow velocity in the middle cerebral artery when sitting is uninterrupted, compared with 2-min light intensity walking breaks every 30 min. The benefit disappeared if the sitting period was prolonged. Zlatar et al. (2019) confirmed that longer sedentary time was significantly associated with lower CBF in lateral and medial frontal regions, which confirmed the earlier work by Bailey et al. (2013) showing that sedentary aging is associated with a longitudinal decline in CBF. Finally, the literature has persistently confirmed the positive impact of regular physical activity on CBF (Kleinloog et al., 2019; Hartman et al., 2020; Maasakkers et al., 2020; Steventon et al., 2020).

White matter hyperintensity volume in the brain is higher with longer sedentary time, and the relationship appears to be modulated by kidney function (Bronas et al., 2019). Rhyu et al. (2010) showed in monkeys that exercise increased the vascular volume fraction in the motor cortex, a response that disappeared after a 3-month sedentary period. Burzynska et al. (2016) reported that moderate to vigorous physical activity was associated with lower white matter hyperintensity volume in healthy older adults, and a randomized controlled trial in older women showed that resistance training reduced the progression in white matter hyperintensity (Bolandzadeh et al., 2015).

The association of sedentary behavior with brain atrophy is also amply evidenced in the literature. Siddarth et al. (2018) reported that the thickness of the medial temporal lobe, the parahippocampal and entorhinal regions and subiculum correlated inversely with hours of sitting per day. Hashimoto et al. (2016) investigated the relationship between physical activity and hippocampal volume and concluded that hippocampal atrophy was associated with physical inactivity. A review by Cooper et al. (2018) confirmed that running improves hippocampal neurogenesis, neural circuitry, and synaptic plasticity. In a sub-sample from the Reykjavik Study cohort, Arnardottir et al. (2016) showed that more gray and white matter volumes at baseline were associated with more total physical activity, and the 5-year change in MRI-derived white matter volume was associated with total physical activity. The authors summarize their findings by confirming that a robust association exists between brain atrophy and lack of physical activity.

## Sleep Insufficiency

Sleep is essential, and enough of it is crucial for cognitive function. The brain uses sleep as an opportunity to rid itself of metabolites that accumulated during wakefulness (Xie et al., 2013), repair connections that may have been damaged (Bellesi et al., 2013), and consolidate learning acquired during the day (Yang et al., 2014).

Many environmental factors can interfere with the adequacy of sleep, including the use of technology (Gamble et al., 2014), the consumption of stimulants (Ogeil and Phillips, 2015), and environmental noise (Hume et al., 2012). Short sleep duration and disturbed sleep, when chronic, are related to several cardiovascular (Huang L.-K. et al., 2020; Huang T. et al., 2020), metabolic and neuropsychiatric diseases (Irwin, 2015). As well, a number of medical conditions can render sleep inadequate, most prominently sleep apnea (Dempsey et al., 2010), and restless leg syndrome (Guo et al., 2017). In studying the impact of sleep disturbances on inflammation or small vessel disease, it is important but often difficult to separate the contribution made by any underlying medical condition from that made by the associated sleep derangement. For example, obstructive sleep apnea does indeed impact sleep adequacy, but it also triggers hypoxia, and it becomes difficult but important to take that into account when studying the impact of sleep on cognition.

With these limitations in mind, there is ample evidence that poor sleep quality is associated with a reduction in cognitive functions (Lim et al., 2013; Osorio et al., 2015). Sleep promotes the formation of dendritic spines to consolidate learning into long-term memory (Yang et al., 2014), and poor sleep quality and duration interrupt this process. Adequate sleep also promotes inflammatory homeostasis and by contrast, insufficient sleep activates several inflammatory mediators and can lead to systemic and chronic low-grade inflammation (Besedovsky et al., 2019). Sleep duration of less than 5 h elevates the body’s inflammatory burden including elevations in CRP, IL-6 and TNFα (Smagula et al., 2016). Disturbed sleep and insomnia are also strongly associated with upregulation of CRP and IL-6 (Irwin et al., 2016). Adolescents are not spared: compared to controls, those suffering from insomnia and short sleep duration exhibit systemic inflammation and significantly higher CRP level (Fernandez-Mendoza et al., 2017; Slavish et al., 2018). In a systematic review of the topic, extreme long sleep duration as well as sleep disturbance and shorter sleep duration were associated with higher levels of CRP and IL-6 (Irwin et al., 2016). Telomere length is an index of cellular aging, and since ongoing inflammation is known to contribute to telomere dysfunction (Jose et al., 2017), it is not surprising that shortened sleep has been shown to contribute to shortening of the telomere (Kaszubowska, 2008; Childs et al., 2015).

Brain atrophy is evident in those who are perennially sleep deprived (Sexton et al., 2014; Gelber et al., 2015). This is presumed to be a consequence of small vessel disease, and several studies have shown an association between poor sleep quality and the severity of white matter hyperintensities noted on MRI (Ramos et al., 2014; Del Brutto et al., 2015). Each hour of reduced sleep duration significantly augmented the expansion rate of the ventricles (Lo et al., 2014). Fjell et al. (2020) correlated self-reported sleep measures with MRI-derived hippocampal volumes in more than 3000 cognitively normal participants and reported that worse sleep characteristics correlated with greater hippocampal volume loss. In restless leg syndrome, a major cause of sleep disturbance, the longer the duration of the condition the more likely it was to be accompanied by brain small vessel disease (Ferri et al., 2016), and obstructive sleep apnea, when moderate to severe, has also been reported as an independent risk factor for the development of cerebral white matter changes (Kim et al., 2013).

The impact of sleep disturbances on CBF has been studied in acute sleep deprivation and in the setting of sleep apnea. Regional cerebral perfusion was reduced after acute sleep deprivation (Zhou et al., 2019), and patients with insomnia showed a consistent pattern of hypoperfusion across 8 pre-selected cerebral regions, with deactivation in the basal ganglia being particularly pronounced (Smith et al., 2002). In untreated obstructive sleep apnea, CBF was significantly lower in multiple brain regions, and the hypoperfusion partially reversed when treatment of the apnea was instituted (Kim et al., 2017). Using SPECT, Joo et al. (2007) showed that in patients suffering with obstructive sleep apnea regional CBF was reduced in bilateral hippocampal gyri and other regions involved in memory function, spatial learning, executive function, and attention. This association of sleep disturbance with reduced CBF in brain regions essential for cognitive functions has subsequently been confirmed (Baril et al., 2015).

## Discussion

The 2020 Lancet Commission Report lists 12 modifiable risk factors that are associated with cognitive decline. The Report states that together these account for 40% of worldwide dementias (Livingston et al., 2020). The report also emphasizes that the number of people with dementia is rising and stresses the importance of considering a life course model for these modifiable risk factors. The report gives the example of weight and blood pressure, which usually fall in later life, and warns that lower weight in later life might signify illness, not an absence of risk.

The three lifestyles chosen for discussion here were included in the Lancet Commission report as contributing to dementia. Their prevalence in society is increasing. Particularly in western society the populations are becoming more obese (Mitchell et al., 2011), more sedentary (Owen et al., 2010), and the demands of work and other responsibilities frequently interfere with the adequacy of sleep (Bixler, 2009). Taking these 3 lifestyles as prototypes for those that contribute to a decline in cognition, a sequence of events is proposed to explain how a harmful lifestyle leads to this unfortunate outcome.

The hypothesis offered suggests that the offending lifestyle leads to the persistent generation of inflammatory mediators that are harmful to the viability of small vessels. Several articles have confirmed the link between small vessel disease and the white matter hyperintensities seen on MRI (Verhaaren et al., 2015; Wardlaw et al., 2015; Rosenberg et al., 2016; ter Telgte et al., 2018). It is proposed that small vessel disease eventually leads to a decline in CBF, followed by atrophy of cerebral structures essential for cognitive function. Our hypothesis does not necessarily imply the activation of neuroinflammation but may lead to it subsequently (Low et al., 2021).

The pathophysiologic sequence of events proposed here is evident with each of the harmful lifestyles selected, but the hypothesis that this sequence of events occurs with every harmful lifestyle will only be confirmed when longitudinal studies make the relevant measurements, in the same population, starting with cognitively normal individuals and following them with simultaneous periodic measurements of cognitive function, inflammation profile, the viability of cerebral small vessels, determination of regional CBF, and measurement of the volume of brain structures relevant to cognitive function. The success of any interventional studies to modify the course of dementia can only be judged against this information.

Ever since the description of the extracellular amyloid plaques and the intracellular tau neurofibrillary tangles accumulating in the brains of Alzheimer patients, the therapeutic emphasis has been on eliminating them from the brain. Several drug trials have now been completed which targeted these proteins without significant favorable impact on the cognitive deficits of the patients (Yiannopoulou et al., 2019). Nonetheless, cerebral amyloidosis and other causes of inflammation can have synergistic deleterious effects on cognitive function. To this date however, the only effective approach to avoiding or delaying dementia may be the correction of lifestyles known to contribute to cognitive decline.

In a major study made possible by the Alzheimer Disease Neuroimaging Initiative (ADNI), which collected information on several brain parameters starting with cognitively normal patients and following them as their cognitive functions declined, it was shown that these proteins accumulated late, following several physiological events in the brain, with the initial trigger to the entire cascade being a decline in CBF, an event that preceded the appearance of the clinical syndrome by years (Iturria-Medina et al., 2016). The hypothesis presented here is further upstream from the decline in CBF and seeks to link lifestyles known to be harmful to cognition into current accepted knowledge.

To explain the link between dementia risk factors and the reduction in CBF, investigators have explored the longitudinal relationship between the presence of cerebral small vessel disease and the decline in CBF, particularly with a view to determining which is cause and which is effect. Shi et al. (2016) performed a systematic review of studies that assessed CBF in small vessel disease, and their results suggest that hypoperfusion in the whole brain and low cortical blood flow is likely a consequence of white matter hyperintensities. Van der Veen et al. (2015) had a year earlier shown that larger periventricular and deep white matter hyperintensities were associated with a decline in CBF 4 years later, but reduced baseline CBF was not associated with progression of lacunes and white matter hyperintensities. The consensus then appears to be that small vessel disease (SVD) precedes the decline in CBF, lending support to the hypothesis proposed here.

Endothelial cells form the main barrier between the circulating blood and the vessel wall, and they are the primary target of inflammation (Steyers and Miller, 2014). Inflammatory markers have been associated with periventricular white matter hyperintensities, the main MRI signature of SVD in the brain (Rosano et al., 2012). Several abnormalities in the interleukin pathways have been linked to SVD including TNFα, IL-10, and IL-21 (Swardfager et al., 2017). Flex et al. (2014) had linked proinflammatory gene polymorphisms to dementia, and C-reactive protein is considered an endothelial toxin that is predictive of SVD (Mitaki et al., 2016). It is therefore no surprise that midlife systemic inflammation has been associated with the appearance of SVD in later life (Walker et al., 2017).

The idea that harmful lifestyles lead to inflammation was emphasized by a number of investigators, most prominently by Littman a number of years ago (Littman et al., 2007), and more recently by Newcombe et al. (2018), and then Nivukoski et al. (2019).

The link between inflammation and subsequent dementia extends to systemic conditions associated with inflammation, including inflammatory bowel disease (Zhang et al., 2020), and rheumatoid arthritis (Sangha et al., 2020), both of which have been associated with a higher dementia risk. This led to several attempts to treat Alzheimer’s disease by targeting neuroinflammation (Ardura-Fabregat et al., 2017), but none were sufficiently promising to merit further assessment.

If the hypothesis offered here is to be truly universal, each of the 12 harmful lifestyles identified in the Lancet Report should be shown to be associated with inflammation and its consequences as proposed here. That is beyond the scope of this review, but inflammation is known to occur in most dementia risk factors identified by the Lancet Report, including excessive alcohol intake, smoking, depression, diabetes, and air pollution. Less education, identified as a risk factor for dementia, may lead to poverty which may lead to SVD through the more affordable pro-inflammatory fat-laden and heavily processed diet.

One cognitive risk factor identified in the Lancet Report that may not at first glance fit into the proposed hypothesis is hearing deficit. Having said that, midlife hearing impairment is associated with temporal lobe volume loss including in the hippocampus (Armstrong et al., 2019). Eckert et al. (2013) have used an estimate of SVD to show that age-related changes in low frequency hearing was related to a global decline in vascular health, and Tabuchi (2014) has described the occurrence of auditory dysfunction in patients with cerebrovascular disease. Hearing deficit may also accompany dementia risk factors such as obesity (Hu et al., 2020), sedentary behavior (Loprinzi, 2013), and sleep apnea (Chopra et al., 2016). Hearing loss therefore may be a consequence of inflammation, and evidence that inflammation-induced vasospasm is involved in the pathogenesis of acquired sensorineural hearing loss was eloquently presented recently by Eisenhut (2019). Windsor and Ruckenstein (2018), in fact suggested that anti-inflammatory therapies may reduce sensorineural hearing loss. In addition, a study by Ponticorvo et al. (2019) showed that there was reduced cerebral perfusion in patients with hearing loss detectable prior to brain structural damage. This is not to deny that the disconnection from the auditory environment that hearing deficit implies may also play a role in inducing cognitive deficit, since those using hearing aids reduce their risk for cognitive decline (Amieva et al., 2018; Maharani et al., 2018), but hearing deficit may fit into the hypothesis presented here better than suspected.

The hypothesis presented raises the need for individual, societal and pharmacological responses that could reduce the risk of dementia. Disseminating the message that dementia is not inevitable as we get older, and that individuals have significant control over their dementia risk, can be very empowering and may lead to individuals incorporating lifestyles and habits that reduce the risk. A concerted effort to spread this message by the organizations implicated in dementia prevention and care can be effective.

It is important in this regard to be reminded that the sequence of pathophysiologic steps identified here are at play for years prior to the appearance of cognitive deficits (Low et al., 2019). Consequently, it is unlikely that dementia would be alleviated by incorporating healthy lifestyles after the appearance of cognitive deficits. For this reason, younger populations should be targeted by the message that we individually have control over our risk for dementia as we age.

It is estimated that the cost of dementia care will overwhelm many healthcare budgets (World Health Organization, 2017). The determination of governments to reduce the prevalence of dementia should lead them to increase public education on the topic, impose taxation on unhealthy dietary drinks and foods, and stand the ground when industry responds. When we became convinced that smoking was the cause of most lung cancers, the healthcare system developed a discourse with the public and with governments which eventually led successfully to limitations on consumption. We need to regenerate that energy to reduce the risk of dementia in society.

Could an anti-inflammatory approach be useful in treating or delaying dementia? This review would suggest that the answer is positive, but clinical trials with anti-inflammatory drugs have found no evidence for efficacy in treating dementia (Rivers-Auty et al., 2020). There are multiple probable reasons for this, in addition to the time displacement between inflammation and its cognitive consequence. Inflammation is complex, and each lifestyle contributing to increased dementia risk may activate one or more specific inflammatory mediators requiring specific inhibitory intervention. Further research into the inflammatory profile associated with each dementia risk factor is needed, followed by the development and testing of an inhibitor. In the meantime, acquisition of healthy lifestyles may be the only path to reducing dementia risk for the individual and prevalence of the condition in society.

## Conculsion

This review proposes a unifying hypothesis that all lifestyles that contribute to cognitive impairment do so in the same stepwise fashion. The harmful lifestyle activates inflammatory mediators which cause cerebral small vessel disease. This then negatively impacts blood flow to the affected region causing it to lose volume and reducing its contribution to cognitive function. The review confirms this sequence in 3 lifestyles: obesity, sedentary existence and sleep insufficiency. It then shows that the same process likely occurs in hearing deficit. If confirmed, this hypothesis suggests individual, societal and therapeutic approaches that may counteract the growing burden of cognitive decline.

## Author Contributions

AH reviewed the literature and wrote the article.

## Conflict of Interest

The author declares that the research was conducted in the absence of any commercial or financial relationships that could be construed as a potential conflict of interest.
